# A key role for stimulus-specific updating of the sensory cortices in the learning of stimulus–reward associations

**DOI:** 10.1093/scan/nsy116

**Published:** 2018-12-20

**Authors:** Berry van den Berg, Benjamin R Geib, Rene San Martín, Marty G Woldorff

**Affiliations:** 1 Center for Cognitive Neuroscience, Duke University, Durham, NC, United States; 2 Department of Psychiatry and Behavioral Sciences, Duke University, Durham, NC, United States; 3 Department of Experimental Psychology, Faculty of Behavioural and Social Sciences, University of Groningen, Groningen, The Netherlands; 4 Department of Social Psychology, Faculty of Behavioural and Social Sciences, University of Groningen, Groningen, The Netherlands; 5 Centro de Neuroeconomia, Universidad Diego Portales, Santiago, Chile

**Keywords:** EEG, FRN, P3, oscillatory alpha, reward learning, decision-making

## Abstract

Successful adaptive behavior requires the learning of associations between stimulus-specific choices and rewarding outcomes. Most research on the mechanisms underlying such processes has focused on subcortical reward-processing regions, in conjunction with frontal circuits. Given the extensive stimulus-specific coding in the sensory cortices, we hypothesized they would play a key role in the learning of stimulus-specific reward associations. We recorded electrical brain activity (using electroencephalogram) during a learning-based decision-making gambling task where, on each trial, participants chose between a face and a house and then received feedback (gain or loss). Within each 20-trial set, either faces or houses were more likely to predict a gain. Results showed that early feedback processing (~200–1200 ms) was independent of the choice made. In contrast, later feedback processing (~1400–1800 ms) was stimulus-specific, reflected by decreased alpha power (reflecting increased cortical activity) over face-selective regions, for winning-*vs*-losing after a face choice but not after a house choice. Finally, as the reward association was learned in a set, there was an increasingly stronger attentional bias towards the more likely winning stimulus, reflected by increasing attentional orienting–related brain activity and increasing likelihood of choosing that stimulus. These results delineate the processes underlying the updating of stimulus–reward associations during feedback-guided learning, which then guide future attentional allocation and decision-making.

## Introduction

To achieve successful adaptive behavior, humans and other animals need to learn to associate specific stimuli and choices with potential rewards, a process that requires the continuous monitoring and incorporation of feedback information. Learning such associations could facilitate predicting whether one will like a new food product based on previous experience with similar foods or whether walking or taking public transportation is a quicker option to get home from work at certain times of the day. Choosing adaptively in probabilistic settings requires an organism to learn, store and continuously update stimulus–reward associations to be available to guide future behavior (e.g. Anderson, 2017).

Previous research investigating the neural processes underlying probabilistic learning has shown an important role for subcortical reward regions, such as the ventral tegmental area (VTA)/substantia nigra (Schultz *et al.*, 1997; Schultz, 2016) and nucleus accumbens (NAcc; Alexander and Crutcher, 1990; Floresco, 2015), in conjunction with frontal regions, such as the anterior cingulate cortex (ACC; Bush *et al.*, 2002; Botvinick, 2007; Klein-Flugge *et al.*, 2016) and orbital frontal cortex (FitzGerald *et al.*, 2009; Sul *et al.*, 2010; Wallis, 2011; Leong *et al.*, 2017), in the processing of value and feedback-related information. Activation in these brain regions has been found to vary according to the difference in value between the actual and the expected outcome (i.e. reward-prediction error), as shown both in humans with functional magnetic resonance imaging (fMRI) and in non-human primates with single-unit recording (Schultz *et al.*, 1997; Niv, 2009).

In addition, in humans, brain activity reflecting the evaluation of feedback information has been tracked non-invasively at the scalp with high temporal resolution by using electroencephalogram (EEG) recordings and analyzing the associated event-related potentials (ERPs). This research has consistently found elicitation of the feedback-related negativity (FRN) response, a fronto-central negative-polarity deflection that peaks in the scalp-recorded ERP around 250 ms following feedback and is larger for negative compared to positive outcomes (Miltner *et al.*, 1997; Gehring and Willoughby, 2002). Following the FRN, outcome feedback also elicits a positive-polarity P3 deflection (starting at ~300 ms) that tends to be larger in response to suboptimal outcomes and has been postulated to reflect the general enhancement of cognitive resources in response to a relative loss (Nieuwenhuis *et al.*, 2005; Polich, 2007; O’Connell *et al.*, 2012; San Martín, 2012; San Martín *et al.*, 2013). In addition, the P3 has been reported to be larger when stimulus values have to be updated, and its amplitude has been found to predict future choice adjustments (Fischer and Ullsperger, 2013; San Martín *et al.*, 2013).

The subcortical and frontal regions mentioned above, however, seem to typically respond to loss-*vs*-gain feedback mostly independently of the specific stimulus with which the feedback was associated. Accordingly, our understanding of the neural mechanisms through which feedback information guides the establishment and updating of stimulus–reward associations remains incomplete, particularly with regard to the possible role of the sensory cortices during the learning and updating of stimulus–choice contingencies. More specifically, we hypothesized that feedback-guided stimulus-specific updating processes would modulate the activity in areas in the sensory cortices specifically involved in the processing of the stimuli whose reward associations are currently being learned.

Relatedly, recent studies have reported that attention can be influenced by specific stimuli that have been previously associated with reward. Specifically, stimulus–reward associations have been shown to bias attention to be rapidly oriented towards those stimuli when they appear in a visual scene (Hickey *et al.*, 2010; Krebs *et al.*, 2011b; San Martín *et al.*, 2016). The general idea is that by biasing attention, stimulus–reward associations can potentially improve environmental responsivity, decision-making and other behavior (San Martín *et al.*, 2016); on the other hand, such associations can also misguide attention when they are associated with a task-irrelevant distractor stimulus or feature (Hickey *et al.*, 2010; Krebs *et al.*, 2010; Krebs *et al.*, 2011a; Krebs *et al.*, 2013). In the spatial attention domain, such biases in attentional orienting can be measured by examining the hallmark ERP component known as the N2pc, a lateralized negative-polarity deflection peaking at ~250 ms over posterior cortex contralateral to the direction of an attentional shift (Hickey *et al.*, 2010; Kappenman and Luck, 2012). However, the stimulus-specific neural updating processes involved in feedback-guided learning that create these attentional biases remain poorly understood.

One possibility for such feedback-based updating would be that neurons in sensory cortices that are involved in stimulus-specific processing would be involved in such learning (Folstein *et al.*, 2013). A classic example of stimulus-specific coding is the set of cortical regions that respond selectively to images of faces, which have been delineated by fMRI and electrophysiological measures (Perrett *et al.*, 1992; McCarthy *et al.*, 1997; Puca *et al.*, 1999; Kanwisher and Yovel, 2006). These face-specific regions include the occipital face area (OFA), the fusiform face area (FFA) and the superior temporal sulcus (STS; Gobbini and Haxby, 2007; Pitcher *et al.*, 2011). In addition, in humans, the processing of faces elicits selective scalp-recorded ERPs, with the largest being the N170 (latency 170 ms), a lateral-inferior occipital negative-polarity deflection that is greater for faces compared to other objects, generally thought to reflect increased activation from the lateral face-selective cortical processing regions (Bentin *et al.*, 1996; Rossion and Jacques, 2012). Face-selective activity is typically extracted by comparing responses to face images to responses to images of other objects, most commonly images of houses and other buildings. fMRI studies have shown that house and building images elicit selective activity in the parahippocampal place area (Epstein and Kanwisher, 1998), a medial-inferior temporal brain region, but such stimuli do not seem to produce a very distinctive marker in the ERPs.

Accordingly, here we focused on stimulus-specific cortical activity for faces (extracted by face-image *vs* building-image response contrasts) and its distinctive scalp ERP/EEG markers. The stimulus and cortical specificity of scalp-recorded electrophysiological measures, such as described above for the face-selective N170, was for the ERPs that are extracted by time-locked averaging the EEG. Also extractable from the EEG, however, are the time-locked power changes in oscillatory EEG activity, which can provide an additional useful window into cortical brain processing. In particular, changes in oscillatory EEG activity in the alpha band (8–14 Hz) have been found to index the directionality of cortical activation, in that decreased alpha power arising from a cortical brain region is typically associated with increased cortical activation, and vice versa (van Dijk *et al.*, 2008; Jensen and Mazaheri, 2010; Scheeringa *et al.*, 2012; van den Berg *et al.*, 2014; van den Berg *et al.*, 2016). A classic example of this effect in spatial attention is the relative decrease in occipital alpha contralateral *vs* ipsilateral to a cued direction of attention (Worden *et al.*, 2000; Foxe and Snyder, 2011; Grent-‘t-Jong *et al.*, 2011), inversely paralleling relative lateralized increase in fMRI activity observed under a similar contrast (Kastner *et al.*, 1999; Hopfinger *et al.*, 2000; Grent-‘t-Jong and Woldorff, 2007; Green *et al.*, 2017).

**Fig. 1:**
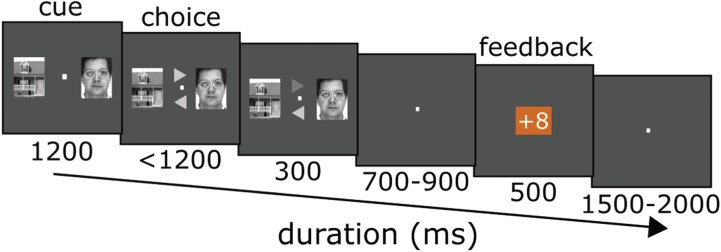
Probabilistic gambling task: each trial started with a dual-object cue stimulus, with a face on one side and a house on the other. Following the cue, participants made a choice of one of these images by selecting the appropriate arrow. Feedback with respect to loss *vs* gain for that choice was then presented.

Here, to investigate the feedback-guided stimulus-specific updating processes, we leveraged the high temporal resolution of EEG, using both ERPs and oscillatory EEG power changes, to track the spatiotemporal cascade of activity over face-processing–selective cortex, while participants performed a probabilistic decision-making gambling task. On each trial (Figure 1), participants were asked to choose between a face and a house, after which they were given feedback indicating that they would receive either a monetary gain or loss on that trial. Within each 20-trial set, either faces or houses were more likely to lead to a gain (probability bias in each set was randomly chosen between 0.50 and 0.75), with the participants instructed to try to learn the likelihood in that set and thereby improve their reward-gaining performance. To identify spatially discernable signals related to face processing, we used a separate localizer task from which we delineated scalp regions that reflect differential processing for faces *vs* houses. Additionally, we analyzed the power changes in oscillatory EEG activity in the alpha band as an inverse index of cortical activations. In particular, modulation in alpha activity was used as a marker for face-selective cortical activation to index the trial-to-trial updating of stimulus-specific reward associations during learning.

## Methods

### Participants

Thirty healthy volunteers [mean age (s.d.): 23.5 (2.9), 18 female, 29 right-handed] participated in the study. All had normal or correct-to-normal vision, and all gave written informed consent. The study was conducted in accordance with protocols approved by the Duke Institutional Review Board. Participants were paid 15 dollars/hour, plus an additional reward-related bonus [mean (sd): 9.6 USD (7.8)]. One participant was excluded due to poor EEG data quality (>40% of the trials rejected due to artifacts), leaving 29 subjects in the final analysis.

### Tasks and stimuli

Participants were asked to do a learning-based decision-making gambling task built upon one we had employed previously (San Martín *et al.*, 2013; San Martín *et al.*, 2016). Stimuli were presented on a 24 inch LCD monitor using the Presentation software package (Neurobehavioral Systems, Inc., Albany, CA). Participants were seated in a comfortable chair with eyes about ~60 cm from the screen.

### Learning-based gambling task

The probability-learning (Plearn) gambling task (Figure 1) consisted of 24 sets of 20 trials each. Within each set, either the faces or houses were more likely to win, with an average probability bias of 0.625 (ranging randomly from 0.50–0.75). As a result, the final probability bias distribution across all sets was roughly Gaussian [mean (sd): 0.625 (0.075)]. Each trial started with the presentation of an image-pair cue (duration 1200 ms), consisting of the image of a face (randomly selected from 20 male faces) and the image of a house (randomly selected from 20 houses), both with a resolution of 72 by 96 pixels. On each trial, the house and face were randomly assigned to appear on either the left or right side of a central fixation. After some delay (1200 ms), a screen for choosing between the two cue stimuli (the choice screen) was then presented, which consisted of two arrows randomly pointing to the left and right, just above and below the fixation, remaining on the screen until a behavioral response was given (up to a maximum of 1200 ms). Participants were instructed to choose the stimulus that they judged would be more likely to lead to a gain by selecting the arrow that pointed in that direction. Participants pressed the top or bottom bumper of a gamepad (Logitech Rumblepad) corresponding to the top or bottom arrow. If no response was given, participants received feedback displaying ‘no response’ on the screen, followed by a loss in points. If a response was made, the chosen arrow was highlighted. Then, following a jittered interval sampled from a uniform distribution between 700 and 900 ms, the gain/loss feedback appeared onscreen (duration 500 ms), which was either a ‘+8’ or a ‘−8’ printed in a blue or orange square (counterbalanced across participants), indicating the valence of the feedback. After the feedback, there was another jittered interval between 1500 and 2000 ms (i.e. 2000–2500 ms following the onset of the feedback stimulus). We intentionally chose a long interval after the feedback to be able to track the stimulus–reward updating neural signals following the processing of the feedback stimulus.

Participants were instructed to try to learn on each set of 20 trials whether faces or houses were more likely to result in a reward in that set and were informed that they could win points, which would translate to real money after the task, by choosing the stimulus (i.e. face or house) that was most likely to win on each set. Each set was followed by feedback as to how many points they had accrued thus far. After a practice period in which the subject demonstrated understanding of how to do the task, the session started with the learning-based decision-making task runs. After completing these, the participants performed the face-*vs*-house localizer task (see below).

### Localizer task

To extract potential regions of interest (ROIs) for assessing the updating in the Plearn paradigm of the face-responsive sensory cortices, we used a face/house localizer task. This task consisted of presenting 480 faces and houses to the participants, which were sampled randomly from the same set of 20 faces and 20 houses that were used in the learning-based decision-making task. Twenty percent of these stimuli were presented as a blurred version, which served as infrequent targets, while the rest were clear. Blurry faces and houses were created by means of convolving a symmetric two-dimensional Gaussian kernel (with a 10-pixel width and height) with the face and house images. The participants’ task was to detect the blurry images and indicate whether they were a face or a house by pressing a button on the response pad (left button for a blurry face and right button for a blurry house). Only the 80% of trials in which a clear face or house was presented (i.e. the non-targets) were used for the EEG analysis. Each face or house was presented for 300 ms, and the next trial started after a jittered interval of 600–800 ms. Twenty-eight participants of the original 30 completed the localizer task. The data from three participants in the localizer task (out of 28) were discarded due to excessive noise (>40% of the trials rejected), leaving 25 participants whose data in this task were used to delineate the face-selective ROIs.

### EEG recording and pre-processing

EEG was DC-recorded [500 Hz sampling rate, with a three-stage cascaded integrator comb low-pass filter (CIC-filter) with a corner frequency of 130 Hz] using a 64-channel, custom-layout, equidistant, extended-coverage electrode cap (Woldorff *et al.*, 2002) and a Brainproducts Actichamp amplifier with active electrodes (Acticap). The data were recorded referenced to the average of all the electrodes. Data were filtered offline using a 0.05 highpass causal finite impulse response (FIR) filter. Independent component (IC) analysis was used to correct for eyeblinks, which were extracted using the extended infomax algorithm as implemented in the EEGlab software package [using EEGlab13.4.4b (Delorme and Makeig, 2004)]. ICs that reflected eyeblinks (one or two ICs per participant) were removed from the data.

For the Plearn task, epochs were extracted from −1000 ms before until 2000 ms after the cue-pair onset and from −1000 ms before until 3500 ms after the onset of the feedback stimulus. For the localizer task, epochs were extracted from −500 ms before until 1000 ms after the onset of the face or house image. All epochs were baseline corrected from −200 ms to the onset of the stimulus (either the cue-pair, feedback or face/house image). Epochs containing any remaining artifacts (e.g. horizontal eye movements, muscle noise) were detected using a threshold (±110 μV from −500 to 1000 ms or to 2500 ms, respectively, for the localizer and Plearn task) and a 30 μV step function (from −200 to 500 ms or to 1000 ms, respectively, for the localizer and Plearn task). Such epochs were then excluded from further analysis. For the ERP analysis, but not the time-frequency analysis, an additional low-pass filter (20 Hz, butterworth fourth order filter) was applied to the epoched and averaged ERP Plearn and localizer data.

### Time-frequency analysis

Frequency decomposition was performed on the epoched EEG average-referenced data using multitaper methods as implemented in the analysis software package Fieldtrip (Oostenveld *et al.*, 2011), in which discrete prolate slepian sequences were used to estimate the power in logarithmically spaced frequencies from 3 to 80 Hz. The window widths for the tapers were 3 cycles for 3 Hz, 4 cycles for 4–7 Hz, 5 cycles for 8–14 Hz, 7 cycles for 15–20 Hz, 10 cycles for 21–50 Hz and 15 cycles for 51–80 Hz. Smoothing by means of multitapers was specified as 5 × log_10_ of each frequency. Log_10_-transformed power spectra for each subject were subsequently binned and averaged according to the various conditions (see below). No baseline correction in the frequency domain was performed for the oscillatory power analyses.

### Data binning and averaging

The epoched EEG data and time-frequency power spectra were binned according to feedback (gains and losses) and choice (face and house), which resulted in an average number of trials for each subject [mean (sd)] for face gain [111 (22)], face loss [96 (20)], house gain [108 (23)] and house loss [92 (19)], after rejection of noisy epochs. As expected, due to learning, there were significantly more gain trials than loss trials [*F*(1,28) = 51.1, *P* = 0.0001], especially towards the latter part of each set. On the other hand, there were no significant differences in the number of gain or loss trials for choosing a face *vs* a house [*F*(1,28) = 0.39, *P* = n.s.].

### Analysis of cue-evoked ERP data

In order to calculate the cue-evoked N2pc response for indexing the attentional bias as it was modulated by reward learning across a set, the following procedure was implemented. To calculate the attentional bias as a function of trial number, we extracted cue-pair-evoked activity averaged in bins of two consecutive trials (i.e. trials 1 and 2 were binned together, trials 3 and 4 were binned together, etc.), separately for each side (left, right) and for each experimentally fixed set-winner (e.g. if a face would have a 0.60 probability of winning in a 20-trial set, that would be a face-winner set). The cue-related attentional bias was assayed by standard N2pc contralateral-*vs*-ipsilateral analysis (Luck and Kappenman, 2012), that is, by subtracting the activity in the contralateral channels (relative to the set-winner) minus the ipsilateral channels and then collapsing over the left and right sides.

### Statistical analysis

For statistical analysis, we first calculated the time-locked ERPs and time-frequency responses as a function of the various event types and conditions. Next, the mean ERP component or Alpha-power (8–14 Hz) amplitudes were extracted from specific scalp ROIs and time-windows of interest (TOIs) based on the literature and the localizer task.

The electrodes that were used for each ROI are reported with respect to the extended 10–10 system naming convention. Because the 10–10 montage and our 64-channel equidistant montage do not overlap perfectly, we report here the 10–10 electrode sites that are closest to our electrodes. Most of our sites were within 0.5 cm of a 10–10 site, and for these we have listed that 10–10 site. Several of our sites were within 0.5 to 1 cm of a standard 10–10 site and these we have listed with a prime (e.g. FC1′). Several of our sites were between 1 and 1.5 cm of the closest 10–10 site, and for these, we have added a letter specifying the relative direction to the standard site. Specifically, we added ‘a’ when more anterior, ‘p’ when more posterior, ‘ℓ’ for more lateral and ‘m’ for more medial than the listed 10–10 site. None of our electrode locations was further than 1.5 cm from a standard 10–10 site.

The FRN and P3 were measured from 200 to 300 ms in a fronto-central ROI (Cz, FCz, Fz, FC1′, FC2′, C1a, C2a) and from 400 to 600 ms in a parietal ROI (channels POz, Pz, CPz, P1a, P2a, P1p, P2p; San Martín, 2012; Fischer and Ullsperger, 2013; San Martín *et al.*, 2013). Subsequently, the extracted values were analyzed with a repeated-measures analysis of variance to test for statistical significance (*P* < 0.05) using the R statistical software package (R Core Team, 2015).

For potential alpha effects following feedback, reflecting a possible stimulus–reward cortical-updating process, we chose a period after the feedback-evoked P3 and before the start of the next trial (600–2000 ms following feedback; recall that the next trial started 2000–2500 ms following the onset of the feedback). The specific scalp ROIs for the potential alpha cortical-updating effect were based on the data extracted from the separate localizer task (averaged across participants).

The EEG data collected during the independent localizer task were used to confirm that the stimuli that we used in this study elicited face-*vs*-house activity differences as found in the existing literature and to delineate the specific locations for these effects using the specific stimuli we employed here. To arrive at our localizer-based ROIs, we first visually inspected all posterior electrodes (i.e. over visual cortex) for the ERP differences between the neural responses to face and house images to make sure these effects were representative of previously reported findings, including the hallmark, face-selective, lateral inferior occipital N170 response. Next we used the set of electrodes that were around the temporal and topographical peaks of these distinctive face–house activity differences in the localizer task to use as the scalp ROIs we would analyze in the Plearn learning task to examine the hypothesized occurrence of cortically specific modulations related to learning, as would be reflected by the post-feedback changes in alpha-band oscillatory power in the Plearn task.

Statistical testing of alpha power was performed using a cluster-based permutation testing approach (Maris and Oostenveld, 2007). *T*-tests were performed on the average power in the alpha band (8–14 Hz) and on each time point within the 600 to 2000 ms TOI separately for each ROI [i.e. the interval following the P3 activity and preceding the next trial (San Martín *et al.*, 2013)]; if the resulting statistic exceeded a *P*-value of 0.05, then that time point was included into a cluster that was formed by including significant adjacent points. To extract the *t*-value relating to the interaction term between the choice and feedback, we subtracted the following conditions: (face gain minus face loss) minus (house gain minus house loss). Cluster statistics were obtained by summing all *t*-values within a cluster. Statistical significance of a cluster was obtained by comparing the cluster statistic to a permutation distribution (created from 1000 iterations by randomly switching the labels of conditions at subject level) and was considered significant at *P* < 0.05 and reported at a trend level if *P* < 0.1.

The cue-related attentional bias (N2pc) was measured from 200 to 300 ms after onset of the cue image pair, from corresponding left and right occipital ROIs [channels (L/R): O1′/O2′, PO3/PO4, PO7′/PO8′, P5i/P6i; Hickey *et al.*, 2010; Kappenman and Luck, 2012; San Martín *et al.*, 2016). Subsequently, we calculated the difference in voltage contralateral *vs* ipsilateral relative to the side on which the set-winner was presented.

To calculate the behavioral learning rate and neural attentional bias, we employed, on a trial-by-trial number basis, a mixed-modeling approach using the *lme4* statistical package (Bates *et al.*, 2015). A varying slope of condition per subject (the random effect) was included in the model if the Akaike Information Criterion (a measure of the quality of the model) improved. To obtain statistical significance, the degrees of freedom were approximated using the Satterwaithe approximation of degrees of freedom as given by the R package *lmertest* (Kuznetsova *et al.*, 2017)*. S*tatistical tests were considered significant at *P* < 0.05.

1) $ proportion\ choosing\  set-{winner}_n={\beta}_{j\left[n\right]}^0+{\beta}^1. trial{\#}_{j\left[n\right]}+{\beta}^2. trial{\#_n}^2+{\beta}^3.{chose}_n+{\beta}^4. trial{\#}_n\times {chose}_n+{\beta}^5.t rial{\#_n}^2\times {chose}_n+{\epsilon}_n $
2) ${attentional\ bias}_n={\beta}_{j\left[n\right]}^0+\kern0.5em {\beta}^1. trial{\#}_{j\left[n\right]}+{\epsilon}_n $

In formula (1), the dependent variable ‘proportion’ reflects the proportion of choosing the winner on each trial#_n_ (1–20) and the predictor variables consisted of an intercept, the first and second degree polynomials for trial# (1–20 and 1:20 squared) and chosen stimulus (face *vs* house). The polynomial term was included in the model to account for non-linearities in the learning rate, notably that subjects’ performance gradually increases early on in the block before tending to asymptote when the most likely set-winner had been learned. In formula (2), the attentional bias (in μV) reflected by the N2pc was estimated by trial number (binned according to the average across two consecutive trials to achieve better signal to noise). For both the behavioral learning rate and neural attentional bias, the final regression model allowed for a varying intercept and a varying slope of trial number for each participant (as indicated by *j*).

Although we did analyze the choice behavior separately for when participants chose house or face, we did not consider choice behavior when analyzing the neural N2pc. The reason for this is that if one calculates the N2pc (and its changes with learning across a block) separately for when faces *vs* houses were the set-winner, the lateralized attention-related N2pc activity could be conflated with lateralized sensory differences between faces and houses (including possible lateralized face-selective N170 activity). However, by collapsing across face and house set-winner blocks, we were able to assess the attentional orienting activity independent of any lateralized sensory activity differences.

## Results

### Localizer task: three ROIs

The ERP difference waves between images of faces and houses in the localizer task showed an early midline occipital positive deflection that was larger for faces compared to houses and largest over the midline electrodes (around Oz and POz), peaking at around 110 ms. This face-selective early occipital positive deflection has been described in previous studies (Eimer, 1998; Itier and Taylor, 2004). Following this midline occipital positivity, faces *vs* houses elicited the large, hallmark, face-selective N170 response (Bentin *et al.*, 1996; Rossion and Jacques, 2012), which started at ~140 ms as a differential, bilateral, lateral-inferior occipital negativity (although slightly larger on the right, as has been typical) and peaking over the lateral inferior sites (PO9ℓ and PO10ℓ) at around 160 ms and lasting until around 200 ms. Shortly later, peaking at around 220 ms, we observed a second smaller more superior bilateral negative-polarity deflection (P1p and PO3, P2p and PO4) that was greater for faces compared to houses. Lastly, after 300 ms, there was a temporally extended activation over both the lateral inferior and superior occipital scalp regions that showed a negative-polarity difference between faces and houses. Observations of such a slower, longer-latency face-selective effect have also been reported previously (e.g. Harris *et al.*, 2011).

**Fig. 2:**
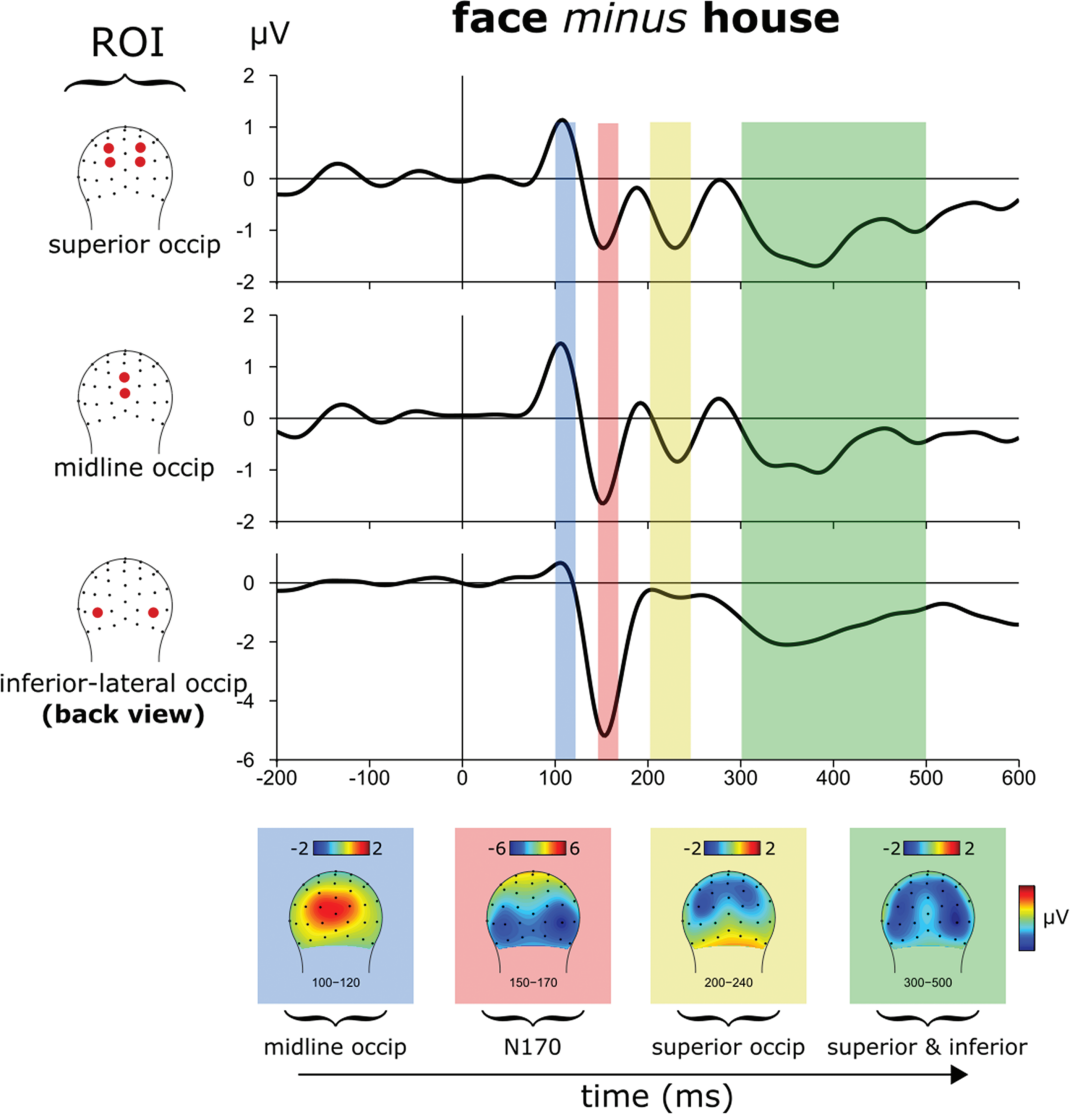
Face-*vs*-house activity from the localizer task: differential activity for faces *vs* houses included the hallmark N170 negative-polarity response over ventrolateral visual cortex (for face responses minus house responses), which was preceded by a midline occipital modulation, and followed by a more superior occipital activation.

Based on these observations of face-selective activity in our localizer task, we defined three non-overlapping ROIs: (i) an early midline occipital ROI (consisting of Oz and POz; Figure 2); (ii) the hallmark, inferior-lateral-occipital face-selective ROI for the N170 (consisting of inferior-lateral-occipital electrodes PO9ℓ and PO10ℓ); (iii) and a superior bilateral one capturing the more superior occipital effect (P1p and PO3, P2p and PO4). These ROIs could potentially reflect activation from the OFA, FFA and the STS (Pitcher *et al.*, 2011), but due to the coarse spatial resolution of EEG, we cannot make firm conclusions about the underlying sources of these effects. More importantly, however, the localizer results confirmed that our stimuli evoked hallmark patterns of face-selective activity, including the midline occipital activation and the lateral-inferior occipital ROI (midline positive deflection and the N170). These ROIs, independently delineated by our separate localizer task, were then used to examine for modulations of alpha oscillatory activity related to learning in the Plearn task.

### Behavioral results and the development of attentional biases toward the cue stimulus with higher reward likelihood

**Fig. 3:**
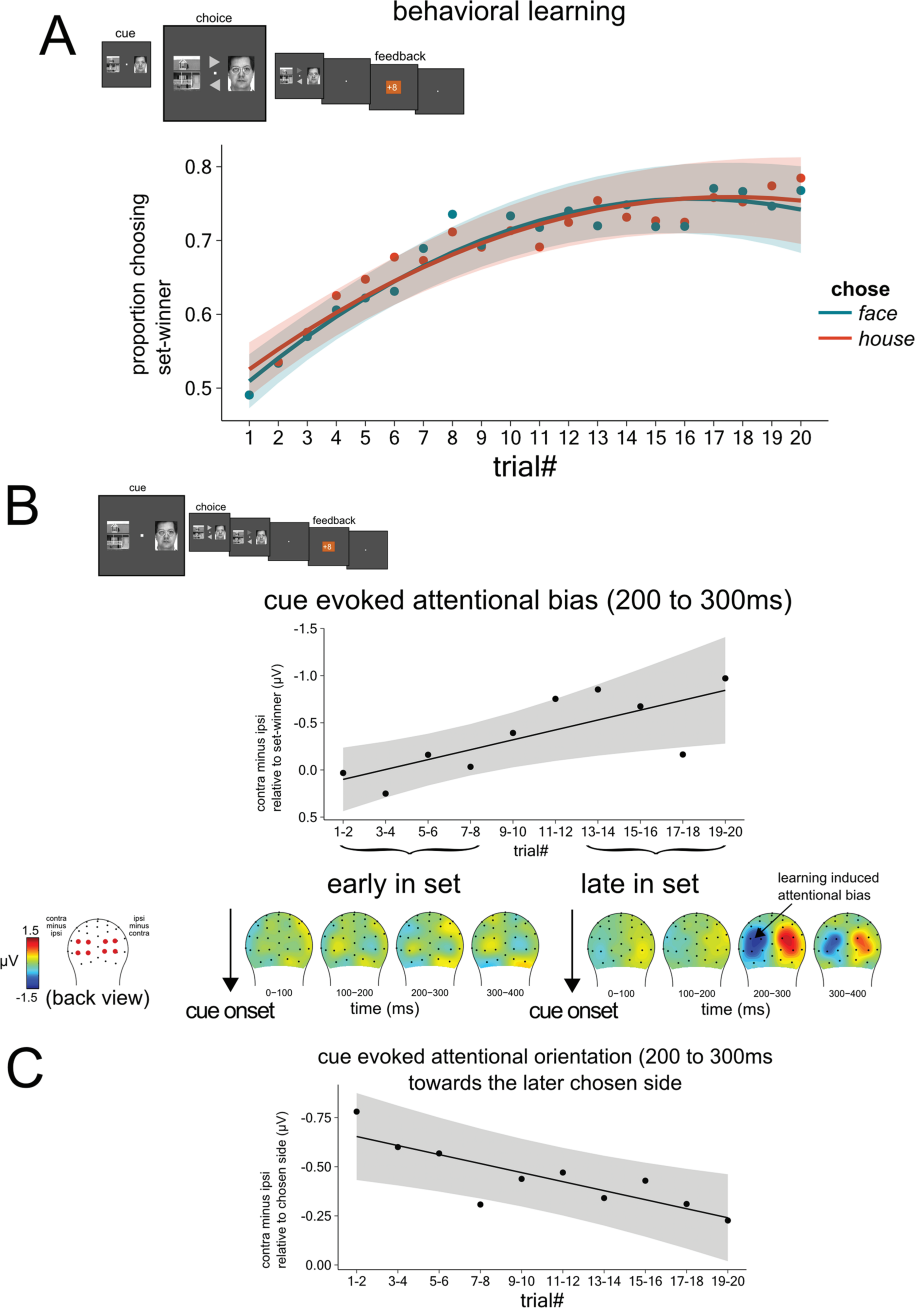
Behavioral and neural learning: during each set of 20 trials, stimulus–reward associations were induced through biasing the probability towards either the face or house, with the probability bias ranging across blocks from 0.50 and 0.75. **(A)** As reflected by the increase in proportion of choosing the probabilistic set-winner across the 20 trials of each set, participants learned whether either the face or the house was more likely to yield a gain. Note that there was no observable difference in the learning curve for whether the set-winner was face or house. **(B)** Neurally, learning was reflected by an increasing amplitude of the attention-sensitive, lateralized negative deflection (the N2pc) contralateral to the set-winner in the cue-pair presentation. This attention-sensitive lateralized ERP activity increased in amplitude as a function of learning across the 20-trial set. **(C)** The size of the N2pc relative to the to-be-chosen side later on that trial across the 20-trial set. The size of the choice-related N2pc reduced in size across the 20-trial block, suggesting that across the block, the N2pc became less strongly driven by the choice that will later be made on the trial and more by the learned reward association. Shaded area represents 95% confidence interval of the model fit.

The behavioral results from the Plearn task (Figure 3A and Table 1) showed that, across each set of 20 trials, participants were able to learn whether the face or the house was the more likely winner for that set [main effect trial#: *F*(2,89) = 80, *P* < 0.0001], which was not observably different for faces and houses [main effect chose: *F*(1,1094) = 0.9, *P* = n.s., chose × trial#: *F*(1,1094) = 0.5, *P* = n.s.]. At the beginning of each 20-trial set, the participants chose the most likely winner for that set at chance (0.50), as would be expected. But by the end of the set, this proportion increased to ~0.75, indicating that the participants had utilized the feedback across the set to learn to choose the most likely probability-based winner. In other words, participants increased the probability of receiving gains by choosing faces more often when the set-winner was a face and by choosing houses more often when the set-winner was a house.

**Table 1:** Model estimates for analysis of the behavioral learning rate: $\boldsymbol{proportion}\ \boldsymbol{choosing}\ \boldsymbol{set}-{\boldsymbol{winner}}_{\boldsymbol{n}}={\boldsymbol{\beta}}_{\boldsymbol{j}\Big[\boldsymbol{n}\Big]}^{\mathbf{0}}+
{\boldsymbol{\beta}}^{\mathbf{1}}.\boldsymbol{trial}{\#}_{\boldsymbol{j}\Big[\boldsymbol{n}\Big]}+{\boldsymbol{\beta}}^{\mathbf{2}}.
\boldsymbol{trial}{\#_{\boldsymbol{n}}}^{\mathbf{2}}+{\boldsymbol{\beta}}^{\mathbf{3}}.{\boldsymbol{chose}}_{\boldsymbol{
n}}+{\boldsymbol{\beta}}^{\mathbf{4}}.\boldsymbol{trial}{\#}_{\boldsymbol{n}}\times {\boldsymbol{chose}}_{\boldsymbol{n}}+{\boldsymbol{\beta}}^{\mathbf{5}}.\boldsymbol{t}\boldsymbol{rial}{\#_{\boldsymbol{n}}}^{\mathbf{2}}\times {\boldsymbol{chose}}_{\boldsymbol{n}}+{\boldsymbol{\epsilon}}_{\boldsymbol{n}}$

The levels in each predictor was trial# 1:20 and chose face and house		
Fixed effect	Estimate (SE)	*t*(df), *P*
${\boldsymbol{\beta}}_{\boldsymbol{j}\Big[\boldsymbol{n}\Big]}^{\mathbf{0}}$	0.49 (0.02)	28.4 (54) < 0.001
${\boldsymbol{\beta}}^{\mathbf{1}}.\boldsymbol{trial}{\#}_{\boldsymbol{j}\Big[\boldsymbol{n}\Big]}$	0.32 (0.003)	11.9 (437) < 0.001
${\boldsymbol{\beta}}^{\mathbf{2}}.\boldsymbol{trial}{\#_{\boldsymbol{n}}}^{\mathbf{2}}$	−0.001 (0.0001)	−8.5 (1094) < 0.001
${\boldsymbol{\beta}}^{\mathbf{3}}.{\boldsymbol{chose}}_{\boldsymbol{n}}$ (house)	−0.01 (0.01)	−0.9 (1094) = n.s.
${\boldsymbol{\beta}}^{\mathbf{4}}.\boldsymbol{trial}{\#}_{\boldsymbol{n}}\times {\boldsymbol{chose}}_{\boldsymbol{n}}$(house)	0.002 (0.002)	1.0 (1094) = n.s.
${\boldsymbol{\beta}}^{\mathbf{5}}.\boldsymbol{t}\boldsymbol{rial}{\#_{\boldsymbol{n}}}^{\mathbf{2}}\times {\boldsymbol{chose}}_{\boldsymbol{n}}\ \big(\mathbf{house}\big)$	−0.0001 (0.0001)	−0.9 (1094) = n.s.

Neurally, at the moment of the presentation of the cue stimuli, this learning process was reflected by attention-sensitive lateralized N2pc activity evoked by the cue pair stimulus (Figure 3B). More specifically, in the first half of each 20-trial set, the N2pc showed little or no reflection of attentional orienting toward the probabilistic set winner stimulus type in the cue-pair presentation, but in the second half of the set, there was clearly an attentional bias, as reflected by a modulation of the lateralized orienting-related N2pc contralateral to the set winner. That is, consistent with previous studies showing with these neural measures the attentional orienting toward reward-associated stimuli (Hickey *et al.*, 2010; San Martín *et al.*, 2016), during the learning of the reward-association here, a negative deflection (200–300 ms) that increased in size over the course of each set was elicited contralateral *vs* ipsilateral to the object in the cue-pair stimulus that predicted a higher probability of reward [*F*(1,106) = 7.1, *P* = 0.009].

In addition, in order to shed light on the link between the N2pc and the choice regardless of whether or not learning had been consolidated, we analyzed the effect of choice on the N2pc over the course of the 20-trial set. Note that we collapsed over whether the chosen image was that of a face or a house to increase signal-to-noise ratio and because of potential sensory differences in the processing of these images. We analyzed the relationship between the N2pc elicited by the cue-pair stimulus relative to the choice made later in the trial, regardless of what the set-winner was (Figure 3C). Results showed a contralateral negative deflection after the presentation of the cue-pair stimulus that was present at the start of the 20-trial set [intercept: −0.65 μV, *t*(100) = −5.9, *P* < 0.001], but which reduced in size over the course of the set [effect of trial#: 0.023 μV per trial, *t*(260) = 2.9, *P* = 0.004] to an amplitude of −0.24 μV relative to the later chosen side.

### Feedback processing

#### ERP results: evaluation of losses *vs* gains

**Fig. 4:**
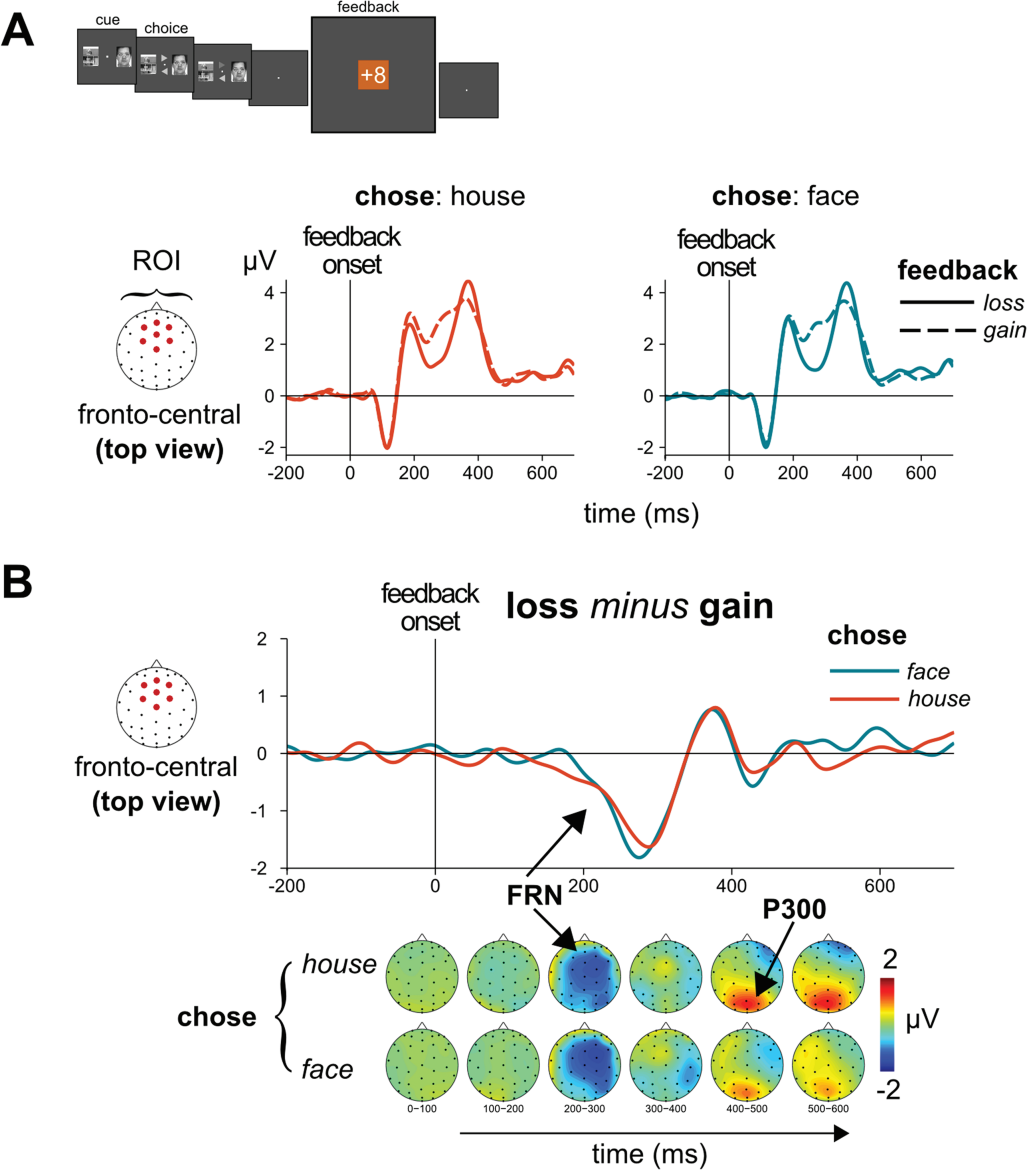
Generic feedback-elicited evaluation processes: **(A)** the grand ERPs to the feedback stimulus (grand averaged across subjects) for when the participant had chosen a face or a house prior to receiving feedback concerning that choice. (**B)** Different waves and topographies reveal the feedback-valence registration as reflected by the feedback-related ERP components (FRN and P3 response following feedback).The classic FRN loss-*vs*-gain effects showed little or no difference as a function of when the stimulus chosen that trial had been a face or a house.

ERP analysis of feedback processing focused on the feedback-evoked potentials and, more precisely, the FRN and the P3. Based on previous literature, we anticipated processing of losses *vs* gains would be reflected by a larger FRN, with the later P3 wave also being larger following losses. We analyzed the mean amplitude in the specified ROIs and TOIs for feedback (loss and gain) and the choice prior to receiving feedback (face and house). The feedback-elicited ERPs confirmed these hypotheses (Figure 4A and B). Following the feedback stimulus, the first difference between losses and gains manifested itself as the hallmark, fronto-central, negative-polarity FRN deflection from around 200 to 300 ms and peaking around 250 ms [*F*(1,28) = 30.1, *P* < 0.0001]. Additionally, there was no observable difference in the amplitude of the FRN as a function of whether the participant had chosen a face as compared to a house on a trial, confirming that the FRN was sensitive to a loss *vs* a gain and not to the particular stimulus type that had been chosen [feedback × choice: *F*(1,28) = 0.3, *P* = n.s.]. Similarly, the P3 was larger for losses compared to gains, as has previously been reported (San Martín *et al.*, 2013) [*F*(1,28) = 22.5, *P* < 0.0001]. In contrast to the FRN, the loss-*vs*-gain effect for the feedback-elicited P3 did show some difference as a function of the stimulus type that had been chosen [feedback × choice: *F*(1,28) = 7.7, *P* = 0.01]. Specifically, contrasts with respect to this interaction indicated that the P3 following losses was somewhat larger when participants had chosen a house *vs* a face [*t*(28) = 3.0, *P* = 0.006; 4.50 μV when choosing a house as compared to 4.02 μV when choosing a face], with no difference for these choices following a gain [*t*(28) = −0.1, *P* = n.s.; ~3.22 μV for both].

#### Oscillatory EEG results: updating of stimulus–reward associations

**Fig. 5:**
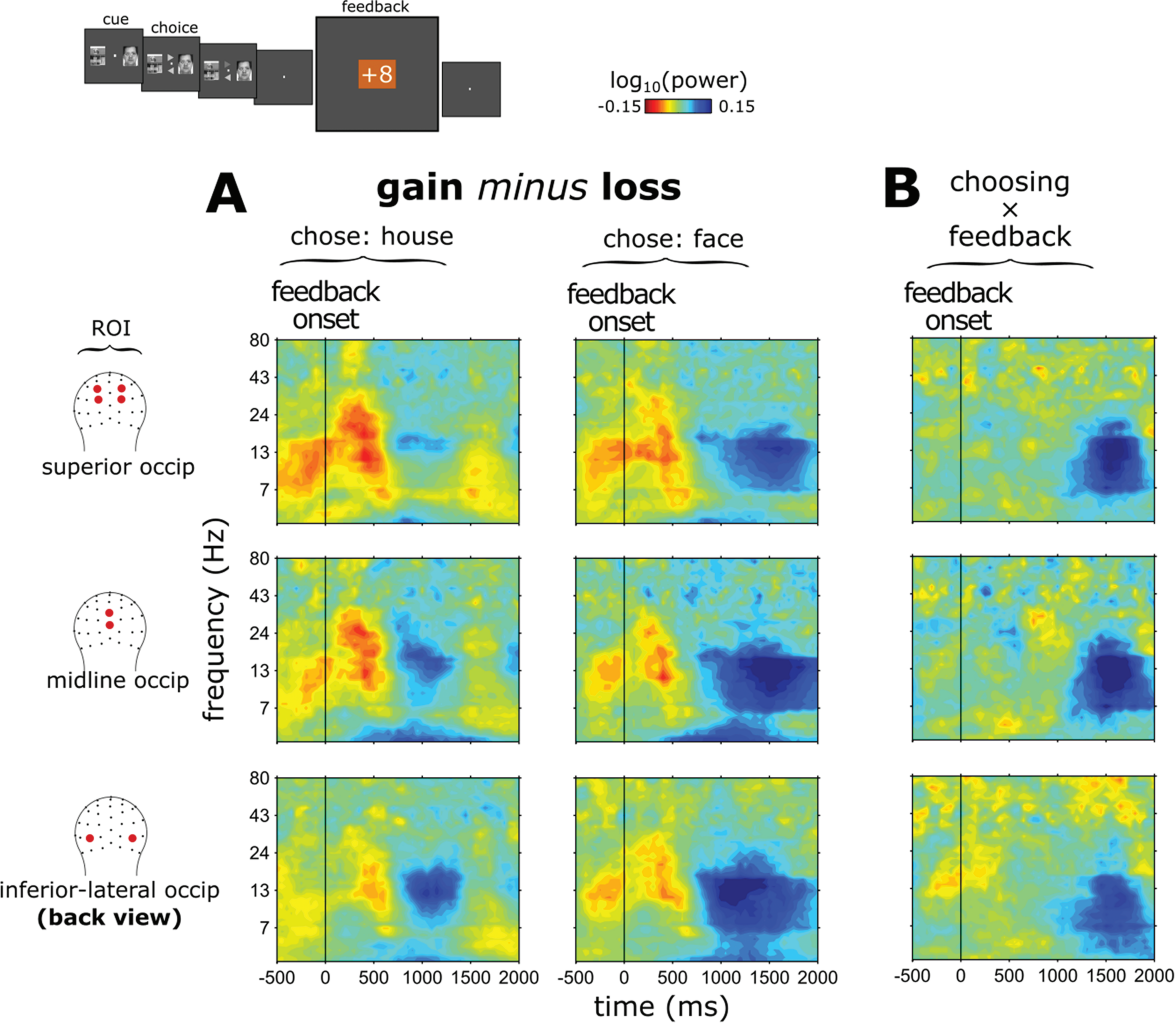
Stimulus-specific feedback-related processes—frequency spectra: **(A)** cortical updating of stimulus–reward associations: time-frequency plots of the differential activity to the feedback stimulus following gains *vs* losses (note the subtraction direction here) over occipital channels in the face ROIs (previously delineated by the localizer task), shown separately following having chosen a face *vs* having chosen a house. The plots reveal a difference in alpha power in these ROIs between 800 and 1800 ms following the feedback for gains *vs* losses, with the later part of this effect being significantly larger when participants had chosen a face as compared to a house. **(B)** This late, cortically specific, alpha-power effect over the face areas can be seen more directly in the double subtraction (interaction) of activity, namely gains-minus-losses for faces minus gains-minus-losses for houses.

**Fig. 6:**
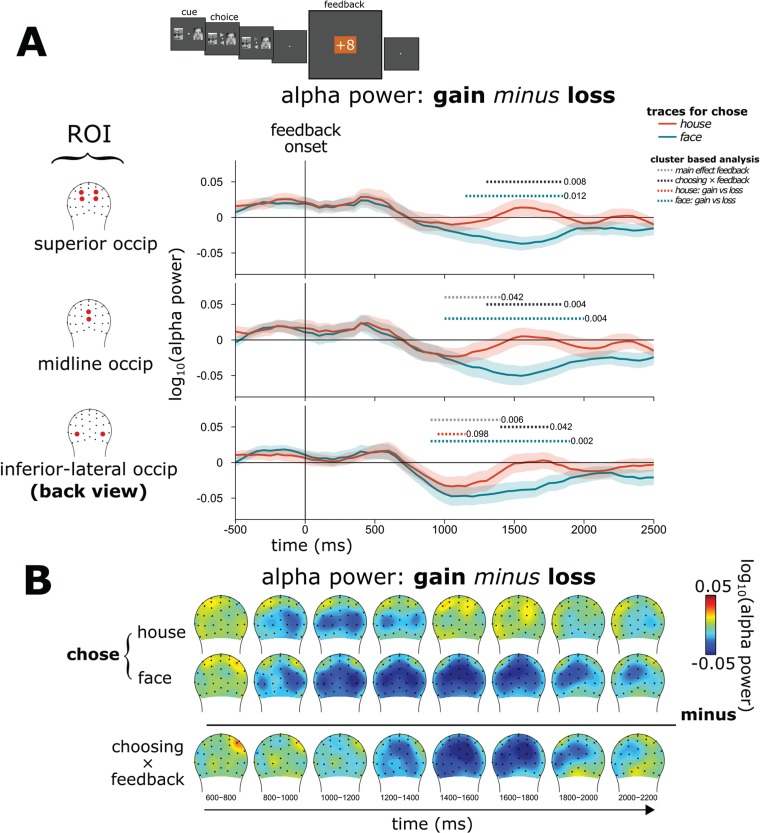
Stimulus-specific feedback-related processes—alpha power: **(A)** overall alpha-power traces show a stimulus-related decrease in power followed by an increase. Cluster-based analysis revealed statistical significance for various contrasts. Shaded area represents mean ± SEM. **(B)** ROI-specific alpha-power traces following gain minus loss feedback. The early part of this long-latency alpha modulation was stimulus–non-specific (no difference for whether a face *vs* a house had been chosen on that trial). Later, the alpha activity showed strong stimulus-specific differences between gain and loss trials as a function of whether a face *vs* a house had been chose on that trial.

We had hypothesized that, following the generic (i.e. non-stimulus–specific) feedback processing (i.e. reflected by the FRN and, to a lesser extent, the P3), a more stimulus-specific cortical signal, as measured by means of oscillatory alpha power, would reflect processes related to the updating of specific stimulus–reward associations in sensory regions in posterior cortex. Specifically, we analyzed potential changes in alpha power over time using cluster-based analysis in the face-specific ROIs defined by the localizer task. Figure 5 shows the frequency spectra for gains minus losses following feedback for when participants had chosen a face *vs* when they had chosen a house, along with the corresponding interaction effect (double difference wave). The frequency spectra in Figure 5 (corresponding to the alpha-power traces in Figure 6A**)** and the alpha-power topographical distributions (Figure 6B) indicate that there was lower amplitude alpha (starting ~900 ms after feedback onset) following feedback of a gain as compared to a loss, both for when a face had been chosen and when a house had been chosen. These cluster-based analyses showed that during the initial part of this time frame (~900–1400 ms following feedback), participants had lower alpha power following gains as compared to losses within the inferior occipital ROI and occipital midline ROI but not the superior occipital ROI (main effect of feedback: **inferior-lateral occipital**: 900–1400 ms, *P* = 0.006; **occipital midline**: 1000–1400 ms, *P* = 0.042; **superior occipital**: no significant clusters found). In this earlier time period, however, significant interactions with the stimulus type that had been chosen were not observed.

**Table 2:** Cluster-based statistics related to the updating of stimulus-specific–reward associations in the alpha (8–14 Hz) frequency range

ROI timewindow, *P*						
	Inferior-lateral occipital		Midline occipital		Superior occipital	
Choosing × feedback	1400–1750 ms	*P* = 0.042	1300–1850 ms	*P* = 0.004	1400–1750 ms	*P* = 0.008
Chose house: gain *vs* loss	950–1150 ms	*P* = 0.098	—	No sig. clusters	—	No sig. clusters
Chose face: gain *vs* loss	900–1900 ms	*P* < 0.002	1000–2000 ms	*P* = 0.004	1150–1850 ms	*P* = 0.012

In contrast, the longer-latency alpha effect over the face-selective ROIs following the feedback was strikingly larger and lasted longer when participants had chosen a face *vs* a house. More specifically, topographical plots of alpha power (Figure 6B: **interaction between feedback and choice**) showed clear stimulus-related specificity for feedback processing over the face-selective ROIs delineated by the localizer task, suggesting the instantiation of stimulus-specific neural activity during feedback processing in these face-specific cortical regions. Specifically, for activity during this later post-feedback time period (but well before the next cue stimulus pair), we observed lower alpha power (i.e. higher cortical activation) for gains as compared to losses when the participant had chosen a face, an effect mostly absent when the participant had chosen a house. This observation was confirmed by cluster-based statistical analysis in the face-specific ROIs as defined by the localizer task (Table 2).

Considering that decreased alpha power in a cortical region typically reflects increased cortical activation (Laufs *et al.*, 2003; Mantini *et al.*, 2007; Scheeringa *et al.*, 2012; Green *et al.*, 2017), this pattern is consistent with the view that there was more cortical activation in the face-processing areas after having chosen a face and received feedback of a gain than after having chosen a house and received feedback of a gain. In sum, the results revealed that, in response to feedback of a gain *vs* a loss, there was an early general (i.e. stimulus-nonspecific) posterior cortical activation followed by a stimulus-specific one. It is important to note that this stimulus-specific alpha modulation following the feedback occurred even though the face/house stimuli had disappeared from view several seconds earlier.

## Discussion

In the present study, we sought to shed light on the neural mechanisms by which stimulus–reward associations might be updated in stimulus-specific cortex. We used a probability-learning gambling task with feedback. On each trial, participants chose between a face and a house stimulus and had to learn across each set of 20 trials which stimulus had a higher probability of gaining a reward in that set. Because face and house stimuli elicit spatially distinctive neural activations in scalp-recorded EEG, we were able to investigate the neural processes related to stimulus-specific feedback processing in the sensory cortices. Behaviorally, the results indicated that participants were able to learn which stimulus yielded a higher probability of reward in each 20-trial set. Neurally, this learning was marked by a cascade of changes in the brain electrical responses. First, feedback evaluation was most quickly reflected at ~250 ms by the FRN, a negative fronto-central deflection, which was then followed by a modulation of the centroparietal P3 (~400 ms), likely reflecting a general increase in cognitive resources. Both of these cortical activations were larger for losses compared to gains. Subsequently (~900–1400 ms after feedback onset), we observed either a decrease (for gains) or increase (for losses) of stimulus–non-specific posterior oscillatory alpha activity that was mostly stimulus–non-specific, but this was then followed by a strongly enhanced stimulus-specific activity for gains compared to losses (~1400–1800 ms) over the sensory face areas when the participant had chosen a face, an effect that was mostly absent when they had chosen a house. Then on the next trial, the learning was further marked by a rapid attentional orienting towards the more-likely-to-be-rewarded stimulus when these stimuli were later presented as a cue pair, an effect that increased across the 20-trial set. These results expand our understanding of the cortical mechanisms by which stimulus-specific regions are activated during feedback learning in service of establishing and/or updating stimulus–reward associations.

Classically, the cascade of neural processes underlying feedback processing has been delineated as starting with feedback evaluation, as reflected by the FRN component for losses relative to gains, and continuing with a general increase in cognitive resources, as reflected by an enhanced P3 wave (Polich, 2007; San Martín, 2012), if the feedback indicated a suboptimal outcome and thus that the choice behavior potentially needs to be adjusted (O’Connell *et al.*, 2012; Fischer and Ullsperger, 2013). These effects were also seen in the present study. We also showed here that the FRN activity was stimulus non-specific, in that it did not differ as a function of whether the feedback was preceded by a face or a house selection. This result is consistent with the idea that the ACC, a likely contributor to the FRN (Gehring and Willoughby, 2002), reflects the detection/evaluation of outcome information independent of the specific stimulus and/or event context (Heilbronner and Hayden, 2016). Although the P3 was slightly larger for losses after choosing a house, compared to choosing a face, it did not differ when the feedback was a gain.

Alpha-power effects began as a general posterior cortical activation, greater for gains compared to losses, but irrespective of whether participants had chosen a face or a house. This effect might be regarded as an increased activation of visual processing regions following gain feedback. Following this general visual cortex activation, between 1400–1800 ms following feedback after having made a face choice, we observed activity over the sensory face regions that was substantially larger following gain feedback compared to loss feedback, with the corresponding effect not being observed following gain-*vs*-loss feedback after participants had chosen a house. Importantly, this stimulus-specific activation occurred even though the face (*vs* house) stimuli had disappeared from the screen seconds before. The activation pattern suggests that these effects, occurring over the occipital-midline, occipital-superior and occipital-inferior face-selective localizer-defined ROIs, potentially reflect activity in the OFA, STS and FFA, which have all been found to selectively process face stimuli (Gobbini and Haxby, 2007; Pitcher *et al.*, 2011). We should mention that the results from the superior-occipital ROI should be interpreted with caution because this activity pattern from the independent localizer task does not appear to have been reported in previous studies. However, because we found this ROI activated in our independent localizer task, we included it in the analysis for completeness.

In the context of these results, we interpret the reduced alpha power over the face ROIs after a gain following a face choice to reflect cortical activation in the face-processing–selective cortical brain regions, as part of the establishing, updating and storage of stimulus–reward associations. Arguably this principle extends to a general mechanism of cortical updating for stimulus–reward association for any stimulus type with respect to feedback. It seems likely that as feedback-guided updating establishes and strengthens stimulus–reward associations, the brain also establishes and strengthens a subsequent reactive attentional bias toward the more-likely-to-be-rewarded stimulus. In our results, this manifested as an enhanced N2pc towards the stimulus that is more likely to win on its next presentation in the cue image pair, paralleling the increased chance that this stimulus would be chosen subsequently at the choice screen. To directly test if changes in alpha power were predictive of changes in the N2pc, we conducted an additional analysis examining the trial-to-trial correlation between the two. The results from this analysis were insufficiently conclusive, perhaps due to a lack of power. Accordingly, the direct trial-to-trial interpretation outlined above warrants further investigation.

The findings of the N2pc being influenced by reward learning are in line with previous studies investigating effects of reward on attentional orienting, which have similarly shown that once a stimulus–reward association has been learned, it tends to induce a larger attentional shift when presented and also predicts the likelihood of subsequent choice behavior (Hickey *et al.*, 2010; Hickey and van Zoest, 2012; San Martín *et al.*, 2016). Our results substantiate these findings by implicating a key role of the sensory cortices in the cascade of processes involved in the formation and neural updating of stimulus–reward associations.

To explore if the attentional bias as marked by the N2pc (at choice-cue presentation) relative to the set-winner was driven by the learned reward association rather than by the increasing likelihood of that choice being made later in the trial, we looked at the effect of trial number on the size of the N2pc relative to the to-be-chosen side. If the N2pc was mainly predictive of the later choice, then the choice-related N2pc should not reduce in size over the course of the set. The results showed an N2pc towards the later chosen side both at the beginning and end of the 20-trial set. However, the size of the choice-related N2pc decreased in size over the course of the set, in contrast to the increase in size of the N2pc across the set towards the rewarding stimulus. This finding supports the interpretation of the N2pc for learned reward association, where the reduction in N2pc for the to-be-chosen side over time suggests that the learned reward association was the stronger factor driving the N2pc rather than just the choice that will be made. We interpret this finding as follows: at the beginning of the set, before the subjects had learned the more rewarding stimulus for that set, their attention and choice behavior was driven by factors other than learned reward association. However, as the reward association was being learned, attention became more and more biased towards the winning feature (either face or house), as shown by an N2pc relative to the learned set-winner later in the 20-trial set. By the end of the set, the choice and bias went hand-in-hand on about 75% of the trials. On the other 25% of the trials, attention still tended to be biased towards the set-winner, but we speculate that participants decided to explore the other option as part of an exploration strategy. In general, it has been shown that participants’ behavioral choices on these types of learning tasks tend to match the probabilities (Herrnstein, 1961; Sugrue *et al.*, 2004). If participants indeed explored the other option following the learned stimulus–reward attentional bias, this behavior would result in a decreasing N2pc towards the chosen side later in the 20-trial set.

In response to stimulus-specific areas being activated following rewarded trials for those stimulus types, the brain seems to then bias attention towards those stimuli. One possibility would be that these stimulus-specific activations reflect a saliency adjustment of the rewarding objects. That is, in addition to coding for stimuli and features, it has been hypothesized that the visual system contains a saliency map for objects and their features (Itti and Koch, 2000; Itti *et al.*, 2001). Such a saliency map could serve as a prioritizing scheme that codes for the importance of various stimuli. By adjusting the saliency of each object category (here, faces or houses), the brain would prioritize the processing of certain objects over other ones. The neuroanatomical properties and location of such saliency maps are under debate, but there is evidence of involvement of sensory visual areas (Li, 2002; Mazer and Gallant, 2003; Torralba *et al.*, 2006; Madden, 2007; Çukur *et al.*, 2013). If indeed the observed cortical activation in response to feedback information is a reflection of saliency map adjustment, then the results from the present study would have implications for understanding the mechanisms by which previously encountered outcome information might be involved in attentional prioritization and cognitive control more generally.

The present study examined mechanisms underlying the learning of reward associations along a relatively short stretch of trials (i.e. each set of 20 trials lasting several minutes), which would then change in the next stretch. Long-term learning in a stable environment would typically lead to value information being updated much more slowly. Notably, learning of object values with different stabilities has been shown to impact activity in the subcortical regions differentially (Kim and Hikosaka, 2013), suggesting different neural mechanisms may be involved based on how stable the learning environment is. Thus, understanding how the processes delineated in this paper differ relative to within a more stable environment would be an important goal for future studies.

In conclusion, we have shown that in response to positive feedback to choices made on specific stimulus types, the brain activates the sensory cortical regions coding for those specific stimulus types. This activation appears to reflect processes related to the establishment and neural updating in stimulus-specific cortical brain regions of the reward associations for those specific stimuli, perhaps by way of enhancing their representation in a cortically based saliency map. Moreover, the establishment and updating of these reward associations in stimulus-specific cortical processing regions during learning is followed in time, on average across trials, by stronger orienting of attention towards those stimuli when they are next encountered. Accordingly, we speculate that the cortical updating in the sensory cortices plays a key role in shaping future choice behavior with respect to those stimuli. We caution that our data cannot establish a direct causal link between these effects, however, as they were shown independently without statistical relations being demonstrated. Future research will need to be performed in order to establish such a causal link.

## Author Contributions

B.v.B., B.R.G, R.S.M and M.G.W designed the study. B.v.B and B.R.G. collected the data. B.v.B analyzed the data. B.v.B., B.R.G, R.S.M and M.G.W wrote the manuscript.
